# Severe Acute Flaccid Myelitis Associated With Enterovirus in Children: Two Phenotypes for Two Evolution Profiles?

**DOI:** 10.3389/fneur.2020.00343

**Published:** 2020-04-28

**Authors:** Melodie Aubart, Cyril Gitiaux, Charles Joris Roux, Raphael Levy, Isabelle Schuffenecker, Audrey Mirand, Nathalie Bach, Florence Moulin, Jean Bergounioux, Marianne Leruez-Ville, Flore Rozenberg, Delphine Sterlin, Lucile Musset, Denise Antona, Nathalie Boddaert, Shen Ying Zhang, Manoelle Kossorotoff, Isabelle Desguerre

**Affiliations:** ^1^Department of Paediatric Neurology, Necker-Enfants malades Hospital, University of Paris, AP-HP, Paris, France; ^2^INSERM 1163, Imagine Institute, Paris, France; ^3^Department of Paediatric Neurophysiology, Necker-Enfants malades Hospital, University of Paris, AP-HP, Paris, France; ^4^INSERM U955-Team 10, Department of Neurosciences, Mondor Biomedical Research Institute, Paris-Est University, Créteil, France; ^5^Department of Paediatric Radiology, Necker-Enfants malades Hospital, University of Paris, AP-HP, Paris, France; ^6^Laboratory of Virology, National Reference Center for Enterovirus, Hôpital de la Croix-Rousse, Hospices Civils de Lyon, Lyon, France; ^7^Laboratory of Virology, National Reference Center for Enterovirus Associated Laboratory, CHU Clermont-Ferrand, Clermont-Ferrand, France; ^8^Paediatric Department, CHU Caen-Normandie, Caen, France; ^9^Intensive Care Unit, Necker-Enfants malades Hospital, University of Paris, AP-HP, Paris, France; ^10^Intensive Care Unit, CHU Raymond Poincaré, Paris Saclay University, AP-HP, Garches, France; ^11^Laboratory of Virology, Necker-Enfants malades Hospital, University of Paris, AP-HP, Paris, France; ^12^Laboratory of Virology, Cochin Hospital, University of Paris, AP-HP, Paris, France; ^13^Laboratory of Immunology, Pitié-Salpétrière Hospital, Sorbonne University, AP-HP, Paris, France; ^14^Direction des maladies infectieuses, Santé publique France, Saint-Maurice, France

**Keywords:** enterovirus, myelitis, children, EV-D68, EV-A71

## Abstract

Acute flaccid myelitis (AFM) is an acute paralysis syndrome defined by a specific inflammation of the anterior horn cells of the spinal cord. From 2014, worrying waves of life-threatening AFM consecutive to enterovirus infection (EV-D68 and EV-A71) have been reported. We describe 10 children displaying an AFM with an EV infection, the treatments performed and the 1 to 3-years follow-up. Two groups of patients were distinguished: 6 children (“*polio-like group*”) had severe motor disability whereas 4 other children (“*brainstem group*”) displayed severe brainstem weakness requiring ventilation support. Electrodiagnostic studies (*n* = 8) support the presence of a motor neuronopathy associated to myelitis. The best prognosis factor seems to be the motor recovery after the first 4 weeks of the disease.

## Introduction

Acute flaccid myelitis (AFM) is a type of acute flaccid paralysis. This type of myelitis has characteristic involvement of the anterior horn cells of the spinal cord (1). The most well-known AFM is poliomyelitis but other EV have benn also involved in poliomyelitis-lke syndromes. EV usually causes fever, respiratory symptoms (cough, asthma), gastro-intestinal symptoms (vomiting, diarrhea, meningitis) and/or skin rash (hand foot and mouth disease). First description of ataxia and flaccid monoparesis with injury of the anterior horn cells followed the first recognized EV-A71 infection in California in 1969 (2) and then the outbreak of EV-A71 in Japan (3) with expression of “poliomyelitis-like disease” (4). In 1999, the Taïwan group described an epidemic of EV-A71 with complications including pulmonary edema, myocarditis, acute flaccid paralysis and/or encephalitis with 78 patients who died (91% were 5-years of age or younger) (5, 6).

Since 2014, some countries have reported waves of AFM in children not associated with poliovirus but with other types of EV such as EV-D68 in the USA, Canada or Japan and EV-A71 in Australia or Spain (7–11). This acute neurological paralysis seems to have some particular features with a lower motor neuron process identified in clinical symptoms, MRI and electromyographic pattern. The initial severity and the poor outcome of the disease was reported in the different series with limited efficacy of the different treatments.

Following other countries, the French EV surveillance network published in 2016 a wave of 59 cases of neurological symptoms associated with EV-A71 and EV-D68 infection in 21(among 196) and 8 (among 274), respectively (12). In Necker-Enfants malades Hospital (Paris, France), 31 children were identified from 2016 to 2018 with neurological features associated with EV infection. They presented with cerebellar ataxia, with or without involvement of cranial nerve, encephalitis and/or myelitis. Among them, 10 cases were AFM with no clinical recovery after the first week of symptoms and treatments. They have in common a particular severity of motor and/or brainstem symptoms and a poor recovery. Here we describe these 10 children, clinical, biological and MRI features, treatments and long-term follow-up after 1- to 3-years.

## Methods

This study was a single-center retrospective review of a group of 10 children who were hospitalized at Necker-Enfants malades Hospital in Paris, France, for AFM between April 1st, 2016 and December 1st, 2018 and diagnosed with a concomitant EV infection within 14 days following neurological symptoms onset.

Definition of AFM was an acute onset of limb weakness, MRI evidence of inflammatory spinal cord lesions and clinical (areflexia and no spasticity) and/or ENMG diagnosis of anterior horn cells of the spinal cord. All children received a clinical examination and clinical presentation was documented with use of the Expanded Disability Status Scale (EDSS) and its functional parameters (13).

All children underwent a brain and a spine MRI in the first days after neurologic symptom onset.

Electrophysiological testing was performed (within the first month of the disease course and controlled after 6–12 months) by pediatric neurologists with experience in pediatric electromyogram studies using a Keypoint device (Natus, Middleton, WI, USA). Each ENMG study included three motor nerves (median, tibial and peroneal) and two sensory nerves (sural and median) conduction studies (NCS). Raw data of sensory and motor NCS were analyzed based on age matched reference values. The needle electromyogram (EMG) protocol consisted in assessing muscles of distal lower limbs (tibialis anterior, extensor digitorum brevis) and muscles of proximal upper limbs (deltoid, biceps brachialis), depending on the clinical motor weakness.

Biological samples were collected at the discretion of the physicians (respiratory tract secretions, cerebrospinal fluid, blood and/or stool samples) and laboratory tests were performed according to institutional protocols. Serum and CSF albumin and Immunoglobulin G (IgG) were assessed by nephelometry (Siemens^TM^ BNII Analyzer) then IgG index were evaluated according to Reiber diagrams (Protis 2 software, Siemens^TM^). Intrathecal IgG synthesis were detected by running both CSF and serum samples on a electrophoresis system.

CSF Viral PCR included HSV, VZV, CMV, EBV and EV.

EV detection was performed by real-time-PCR (ENTEROVIRUS- R-gene®, bioMérieux or Rhino&EV/Cc r-gene®, bioMérieux) at the Necker virology laboratory. Identification of the EV type was obtained by EV-D68 RT-PCR and Sanger sequencing of the viral protein 1 (VP1) or the VP4-VP2 coding genes at the National Reference Center for Enteroviruses in Lyon (France).

Clinical and biological data were collected and assessed by a representative child neurologist (MA) and MRI data were collected and assessed in a blinded protocol by a representative child neuroradiologist (CJR).

Treatments were initiated at the discretion of the physicians: intravenous methylprednisone, oral steroids, intravenous immunoglobulins, therapeutic plasma exchange, Rituximab. Brain and spine MRI and ENMG were performed after 3 to 24 months of follow-up. Informed consents for using of data, photo and video were obtained from all parents of patients according to french requirements.

## Results

### Initial Onset

A total of 10 AFM cases with a documented EV infection were diagnosed in the Necker-Enfants malades Hospital in Paris between 2016 June and 2018 December. The median age at onset was 3.2-years (range, 1–9).

All children had fever before they developed AFM, within a median of 3 days before neurological symptoms (range, 1–7 days).

All patients developed an asymmetrical motor weakness of 1 (*n* = 6), 2 (*n* = 1), or 4 limbs (*n* = 3) with areflexia, no spasticity and fasciculations making the diagnosis of AFM. No patient had bowel or bladder sphincter disorder. Six patients complained of neck or back pain or stiffness.

Two groups of patients were distinguished through their clinical presentation at admission: 6 children (named “*polio-like group*”) had initial asymmetrical severe motor disability and areflexia (1 or more limb) associated or not with transitional cerebellar symptoms whereas 4 other children (named “*brainstem group*”) had initial coma with flaccid tetraplegia and severe brainstem weakness requiring ventilation support. In the *brainstem group*, 2 patients out of 4 first presented with acute severe but reversible myocardial failure.

**Figure 1:**
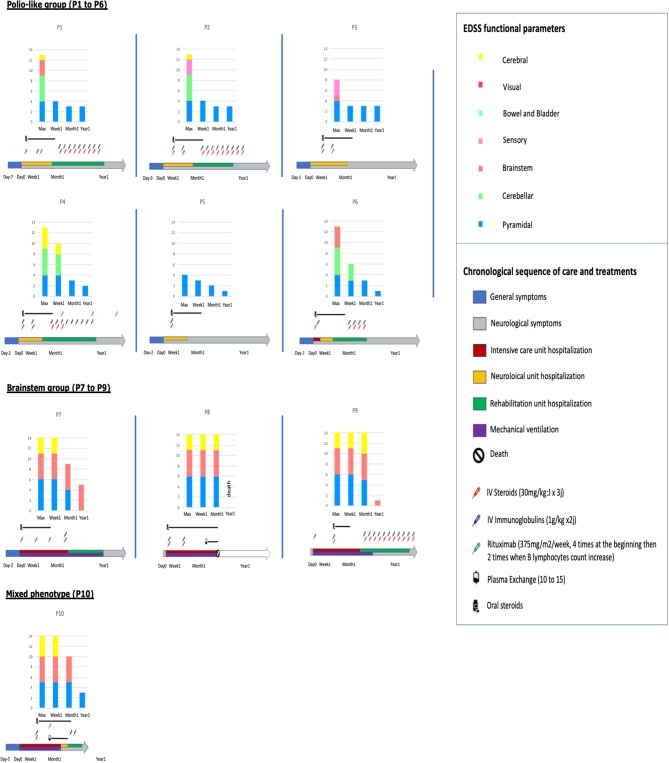
Sequence of care and treatments and evolution of EDSS functional parameters in the 10 patients.

Median total EDSS functional parameters score was 13.1 (4 to 16), consistent with severe functional symptoms. Details are shown in Figure 1 and Supplementary Table 1 summarizes clinical features of the included patients.

### Biological Findings

Standard blood tests (cell counts, hepatic, renal, and inflammatory tests) were done in all patients and showed no anomaly except 4 with a high percentage of neutrophils in the white blood cells.

CSF examination was done in 9/10 patients within 4 to 9 days (median 6 days) after the beginning of the fever and within 1 to 6 days (median 2 days) after neurological symptoms onset. Results are shown in Supplementary Table 2. Pleiocytosis was present in 6 children (median 120 cells/mm^3^, 0 to 220, predominance of lymphocytes or neutrophils), with elevated protein in 7 (median 0.42 g/L, 0.24–0.47). CSF oligoclonal bands were identified in 2/8 patients and a blood-brain barrier dysfunction in 6/8. Interferon-alpha secretion was positive in CSF in 3/5 patients (3–9–25 UI/ml). No difference among biological results was observed between the 2 groups of patients. However, the highest interferon alpha result (25 UI/ml) was obtained in the only tested patient of the *brainstem group*.

CSF viral PCR results were all negative. Six out of 10 nasopharyngeal specimens were positive for EV by PCR. Among stool specimens EV PCR was positive in 8/9. All the negative nasopharyngeal samples were obtained more than 9 days after the beginning of the fever, 10 days for the stool samples. For 8 patients, EV genotyping identified 4 different types of EV: 4 EV-A71 (stool samples), 2 EV-D68 (respiratory sample), 1 CV-B5 (stool sample), 1 CV-A16 (stool sample). For the remaining 2 patients, EV genotyping was not possible.

### Initial Brain and Medullar MR Imaging

**Figure 2:**
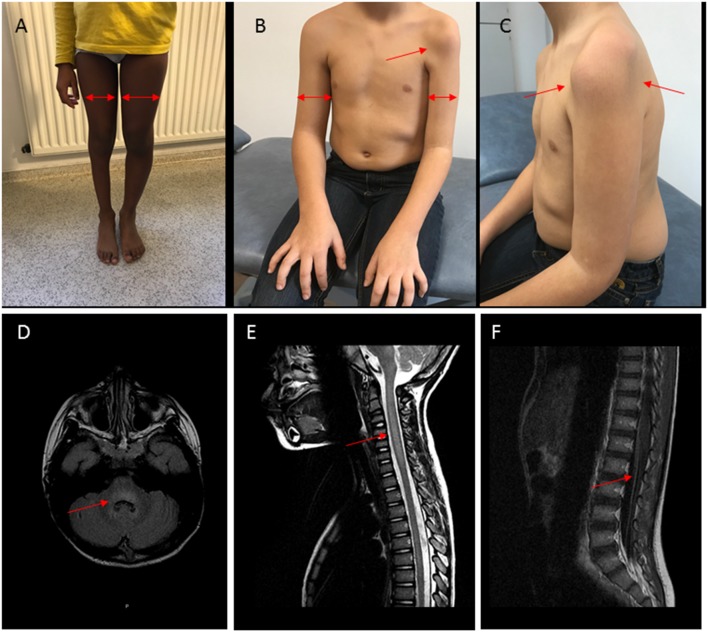
Clinical and MRI findings in patients with AFM and polio-like features. **(A)** Patient P3, right leg monoparesis, 3-years evolution. Proximal and distal muscular atrophy (arrows). **(B,C)** Patient P1, left arm monoparesis, 2-years evolution. Proximal and distal muscular atrophy (arrows). **(D–F)** Patient P3, MRI at the acute stage. **(D)** Hyperintensity (arrow) in T2FLAIR sequence with rhombencephalitis, **(E)** Hyperintensity and edema (arrow) in T2 sequence with myelitis, **(F)** vascular nerve roots enhancement (arrow).

On MR imaging, brainstem lesions were identified in the 10 patients within a median of 4 days (0–14) after the neurological symptom onset. The results are shown in Figure 2 and Supplementary Table 3. The cerebellar peduncles, dentate nucleus of cerebellum, mesencephalon, pons cerebelli, and medulla were the most commonly involved sites, showing an increased T2 and flair intensity and a decreased T1 intensity. Two patients showed associated thalamus involvement and eight patients extensive spinal cord lesions. These spinal cord lesions were T2 central hyperintensity in 2 children and diffuse hyperintensity in the other ones, were not enhanced by contrast agent. Vascular enhancement of nerve roots was observed in 7 children, revealing an MRI motor neuron involvement.

### Electromyogram Findings

Electrodiagnostic studies (*n* = 8) performed within the first month after disease onset, displayed a motor neuronopathy with no motor or sensory abnormalities on nerve conduction studies of affected limbs (n=6/8). Electrophysiological changes were observed in one upper extremity (*n* = 3/6) and in one lower extremity (*n* = 3/6). A more diffuse motor axonal neuropathy was observed for two patients (*n* = 2/8, P2 and P8).

### Treatments

All patients received intravenous pulse of methylprednisolone (MP) (30 mg/m^2^ max 1000 mg × 3 days) and intravenous immunoglobulins (Ig) (1 g/kg × 2 days) within the first week after neurological symptoms onset, excepted P1 (Ig at Day 11), P9 (MP at Day 13), and P10 (MP and Ig at Day 10), (Figure 1). Nine patients out the 10 received iterative MP and/or Ig monthly. Two patients of the *brainstem group* received plasmapheresis: P8 at the 6th week for 17 times and P10 at the 3rd week for 10 times. These 2 patients also received Rituximab (RTX) (375 mg/m^2^/week × 4) as well as one patient of the *polio-like group* (P4).

### Long Term Follow-Up

Long-term evolution of the patients is summarized in Figure 1. Among the 10 children, six required intensive care, four invasive ventilatory support, three a tracheotomy, one died. Motor function improvement was incomplete in 8/10 cases at 12 month follow-up with severe segmental muscular atrophy of one or more limbs with areflexia and fasciculations and long-term rehabilitation.

- In the *polio-like group*, no patients needed ventilatory support and all the brainstem symptoms (in particular cerebellar syndrom) disappeared within the first week after the beginning of treatment (never before treatment). Whereas, 1 year after the onset of the disease, all had a non-zero EDSS pyramidal functional parameter, at 3/6 (severe monoplegia) in 3/6 patients (Figures 1, 2 and Supplementary Videos 1, 2). There is no difference of evolution regarding the treatments excepted P4, the only patient who received RTX who also had the best motor functional improvement among the MP/Ig-non-responder patients of this group.
- In the *brainstem group*, all the patients were hospitalized in intensive care unit and required invasive ventilator support over 1 month. A girl (P8) died from complication of her neurological state at M3 without any improvement (complete quadriplegia, palsy of all cranial nerves and no respiratory or swallowing functions) whereas the 3 other patients required 1, 6, and 12 months of mechanical ventilation and enteral feeding as a consequence of their brainstem deficiency. Neurological recovery was complete for one of them at 1 year of the beginning of the symptoms (EDSS brainstem functional parameter at 1). P10, who presented a combined phenotype *brainstem*/*polio-like* required a mechanical ventilation and enteral feeding for only 1 month. He had a moderate motor weakness of the left shoulder and a no more motor weakness of the right limb with ongoing progress after 1-year. He received MP and Ig at Day 10, plasma exchange at the 3rd week for 10 times and RTX at the same time and at 6 months.

No patient developed iatrogenic adverse events during the care period.

Among the 10 patients, 8 had an MRI follow up between 3 and 6 months after onset. Involvement of brainstem, cerebellar peduncles, dentate nucleus and mesencephalon was still visible (in the form of a T2 hyperintensity more moderate than initially) in 6 patients, spinal cervical cord involvement in 4 patients, and thalamus involvement in 1.

Enhancement of nerve roots had disappeared in all patients.

When reassessed (*n* = 4/8, +3m–+3y after disease onset), electrodiagnostic studies showed persistent decrease in response amplitudes of compound muscle action potentials and/or in recruitment of motor unit potentials on needle EMG, even years after the disease onset.

## Discussion

AFM is a specific clinical phenotype of acute flaccid paralysis associating symptoms of central nervous system involvement (spinal cord, brainstem) and radiculitis with a specific motor neuron involvement, affecting 1 to 4 limbs (1). The cause of AFM (besides poliomyelitis) remains elusive. An infectious agent is only rarely detected in CSF. A growing number of AFM cases has been reported during EV-A71 and EV-D68 outbreaks, initially in Asia since the 1970's and then in the USA and Europe since 2014 with identification of these viruses in stool and respiratory samples from a significant proportion of cases. It suggested a possible pathogenous role of these viruses (5, 6, 9, 11, 14–19). Moreover, murine models added strong arguments in favor of this hypothesis (20). More recently, a US multicentric study demonstrated the presence of antibodies to conserved EV peptides in 22/26 (84%) of AFM pediatric patient compared to 7/50 (14%) non-AFM pediatric patients, raising the question of a common pattern to all enterovirus (21). Following other countries, the French EV surveillance network published in 2016 a wave of 59 cases of neurological symptoms (cerebellar, encephalitis, myelitis) associated with EV infection (12). Among these children, we described here the clinical, biological and MRI features, treatments and long-term follow-up of 10 children with AFM.

The 10 patients had clinical symptoms of AFM: acute flaccid paralysis without pyramidal syndrome but depressed deep reflexes of 1 to 4 limbs and more or less intense brainstem features. All the patients who had an electromyogram displayed a typical diagnosis pattern of motor neuropathy involving anterior horn neuron of the spinal cord. These peripheral features were associated, on MRI, with central damages (T2 hyperintensity of the dorsal pons, dentate nuclei, mesencephalon and medulla and frequent spinal cord injuries). These features were concordant with a diagnosis of rhombencephalitis with radiculomyelitis and were present in all patients, as previously observed (18, 19).

Despite these similarities in diagnosis, we distinguished 2 different groups of patients regarding the clinical presentation at admission and the long term follow up (Figure 1):

- A “*polio-like” group* with minor symptoms of ataxia and/or cranial nerve involvement but severe AFM and long-term motor paralysis of just one limb.
- A “*brainstem” group* with predominance of bulbar symptoms (ophtalmoplegia, swallowing disorder, ventilation support requirement) and a faster improvement of the limb paralysis comparing to the bulbar paralysis.

In the 2 groups, children displayed fever and respiratory and/or gastro-intestinal symptoms around 3 days before neurological symptoms (range 1 to 7 days). Lumbar puncture found a meningitis in 6/9 patients, frequently with neutrophils, but EV PCR was always negative in the CSF. These results are consistent with previously published results. Peripheral samples were positive for EV in stool (8/10) and positive in 6/10 in the respiratory samples. Various types of EV were identified in both subgroups: 4 EV-A71, 2 EV-D68, and 2 others (CV-B5 and CV-A16). Some of the publications described cohorts of patients carrying all an EV-D68 or all an EV-A71(6, 11, 15–17) however others described a cohort of similar AFM patients reported with the presence of different EV strains (9). Although EV-A71 and EV-D68 were identified in 60% of our AFM cases (100% in the *brainstem group*), recent data suggest a more common physiological pattern of various types of EV in AFM patients. This result raises the question of genetic predisposition of an anatomical neurological target of the host to the virus rather than a preferential target of some types of EV.

Clinical evolution of the patients were different between the two subgroups: *polio-like* and *brainstem groups* (Figure 1). These differences in evolution and prognosis have not been reported in previous studies. Patients of the *polio-like group* did not require ventilation support. However, they presented with, a major motor paralysis of one or more limbs with incomplete clinical recovery during the 18 first months of evolution in 4 of the 6 patients (P1 to P4). Thus, the early motor evolution within the first month of the disease seems to be a good indicator of the long-term recovery. The proximal motor recovery seems to be the largest source of long-term motor disability. We noted that clinical improvement can go on more than 1 year. Among the 4 patients with an incomplete motor improvement, we observed no difference in EDSS score between those who received monthly IV steroids and immunoglobulins (P1 and P2) 12 months long and the one who received these treatments only in the first month (P3). However, longitudinal individual evolution seemed to be better in patients with long-term treatment. In this group, one patient (P4) received Rituximab and had the best motor long-term motor recovery at 2-years post-onset (Supplementary Videos 1, 2).

Patients of the *brainstem group* required mechanical ventilation. One patient (P8) died after a decision of limitation of medical treatments. She received Rituximab at week 2 and immune-adsorptions at week 6 but no motor or brainstem improvements were observed after 3 months. P7 and P9 had a complete motor recovery within the first months but needed a long-term ventilation support. P10, the only patient of the group who received therapeutic plasma exchange and Rituximab at week 2, had a short-term ventilation (1 month), a complete motor recovery of the polio-like features of his lower limb but a slow but continue recovery of his left shoulder 12 months after the beginning of the symptoms. These evolutions suggested that initial specific phenotypes seem to have specific evolution profiles whatever the treatments, even if intensive rehabilitation is often required. However, early Rituximab and therapeutic plasma exchanges (with steroids and intravenous immunoglobulins) seem to be well-tolerated. Two patients of the cohort who received these treatments seem to have a better motor recoovery. Yea et al. also reported that these treatments were well-tolerated in a cluster of acute flaccid paralysis. However, stronger and large studies should be conducted to support evidence for these treatments. Moreover, long-term follow-up seems to show ongoing improvement and slow motor recovery until 18 months post-onset in our cohort and in literature, suggesting benefit of a long-term active rehabilitation (9).

Physicians and especially pediatricians should be aware of the specific phenotype of AFM with acute medullar paralysis (with or without involvement of the brainstem) associated with peripheral features (anterior horn cells of the spinal cord/axon). Early collection of respiratory and stool samples is of major importance in this case since, even with a pleiocytosis in the CSF, EV PCR in the CSF is always negative. Two different clinical presentations are observed: poliomyelitis-like and severe brainstem phenotypes. In the *polio-like group*, evolution within the first month of the motor parameters seems to be a good indicator of the long-term recovery even if improvement can further go on for more than 1 year. In the *brainstem group*, bulbar features are life-threatening and ventilation requirement can go for months. Mixed profile of polio-like and brainstem phenotypes can be observed. Long-term ventilation and rehabilitation management in these children should be anticipated in particular during EV-A71 and EV-D68 outbreak periods outbreak periods. More studies are required to better understand if some genetic predispositions make some children more prone to develop severe neurological presentation after an EV infection.

## Data Availability Statement

The datasets generated for this study are available on request to the corresponding author.

## Ethics Statement

Written informed consent was obtained from the minor(s)' legal guardian/next of kin for the publication of any potentially identifiable images or data included in this article.

## Author Contributions

All children received a clinical examination by MA and/or MK. MRI were analyzed by CR and RL. ENMG were performed by CG. Biological laboratory tests were performed by DS, ML-V, FR, and IS. MA organized the database. MA, CG, and CR wrote sections of the manuscript. All authors contributed to manuscript revision, read, and approved the submitted version.

## Conflict of Interest

The authors declare that the research was conducted in the absence of any commercial or financial relationships that could be construed as a potential conflict of interest.

## Supplementary Material

Supplementary Table 1 Clinical presentation and evolution of patients.

Supplementary Table 2 Biological data.

Supplementary Table 3 MRI data.

Supplementary Video 1 Patient P3, right leg monoparesis, 3-years evolution, no treatment since the acute stage.

Supplementary Video 2 Patient P4, left leg monoparesis, 3-years evolution, Intravenous corticosteroids and immunoglobulins by month during a year and Rituximab from the 3rd month to the 18th.
