# Supplementary material for: Severe Acute Flaccid Myelitis Associated With Enterovirus in Children: Two Phenotypes for Two Evolution Profiles?
Source: Front Neurol. 2020 Apr 28;11:343. doi: 10.3389/fneur.2020.00343 (PMC7198806; doi:10.3389/fneur.2020.00343)
Supplement: Supplementary Table 1 — Clinical presentation and evolution of patients. [file Data_Sheet_1.DOCX]

Supplementary data

Supplementary Table 1: Clinical presentation and evolution of patients

Supplementary Table 2: Biological data

Table 3 : MRI data
